# Regulated degradation of KCC2, a potassium-chloride co-transporter required for synaptic transmission and neurodevelopment

**DOI:** 10.1080/19336950.2025.2607247

**Published:** 2025-12-23

**Authors:** Morgan Kok, Elias Aizenman, Christopher J. Guerriero, Jeffrey L. Brodsky

**Affiliations:** a Department of Biological Sciences, University of Pittsburgh, Pittsburgh, PA, USA; b Department of Neurobiology and the Pittsburgh Institute for Neurodegenerative Diseases, University of Pittsburgh School of Medicine, Pittsburgh, PA, USA

**Keywords:** SLC12A5, pathogenicity, protein biogenesis, ER quality control, ERAD, lysosome

## Abstract

Neuronal function requires fine-tuned and coordinated activity of several ion channels and transporters. One member of this ensemble is the KCC2 potassium-chloride cotransporter. Because KCC2 expression is required for GABA-dependent inhibitory synaptic transmission, mutations in the gene encoding KCC2 (*SLC12A5*) have been linked to several diseases that also arise from defects in GABA signaling, including epilepsy, schizophrenia, and autism spectrum disorders. Although characterization of the corresponding mutant proteins is ongoing, KCC2 mutants may reside at the cell surface but lack function, they may remain trapped intracellularly and are thus unable to function at the cell surface, or they may be readily degraded. In this article, we summarize these data and emphasize the importance of protein degradation and protease activity during KCC2 quality control, i.e. the pathway that ensures only properly folded and mature KCC2 can traffic to and function at the cell surface. We also highlight how proteolysis regulates the amount of active KCC2 at the cell surface, i.e. KCC2 quantity control. Finally, because previously unidentified KCC2 mutants are continuously being discovered, we discuss the use of predictive pathogenicity algorithms to provide researchers with information on potential disease outcomes.

## Introduction

**Figure 1.**
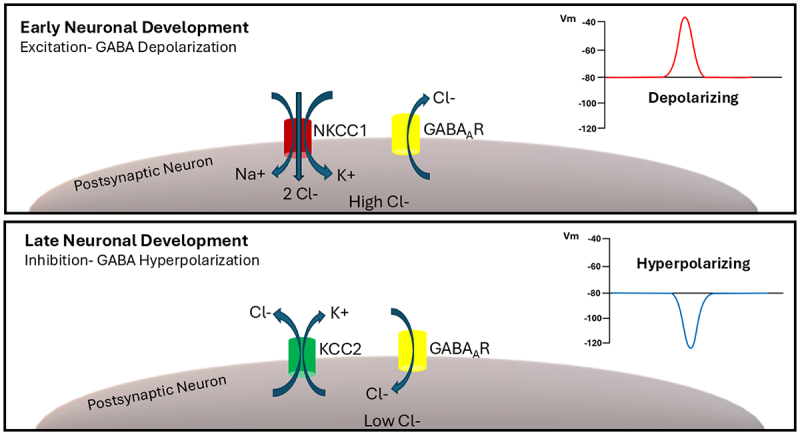
In early neuronal development, intracellular chloride concentrations are high due to the activity of the sodium-potassium-chloride co-transporter 1 (NKCC1). Upon synaptic activation, chloride exits the postsynaptic neuron through GABA receptors. This triggers a depolarization of the plasma membrane, and an excitatory GABA signal is produced. In late neuronal development, KCC2 is expressed and transports potassium and chloride ions out of the cell. As a result, the intracellular chloride concentration is reduced, so chloride enters the postsynaptic neuron through activated GABA receptors. This leads to hyperpolarization of the plasma membrane, and an inhibitory GABA signal is produced.

The potassium-chloride co-transporter 2 (KCC2) is the gatekeeper to synaptic inhibition in the central nervous system. Without its activity, or in case of decreased expression, GABA and glycine become, in effect, excitatory synaptic transmitters [1,2]. KCC2, which is encoded by the *SLC12A5* gene (Solute Carrier Family 12 Member 5; Chromosome 20 - NC_000020.11), is a ~ 130 kDa 12 transmembrane domain-containing protein and transports chloride, as well as potassium, extracellularly from the cytoplasm [3], thus playing a critical role in regulating the intracellular concentrations of these ions. Consequently, this process maintains low internal chloride concentrations, thereby establishing a steep anion gradient that drives chloride into cells after GABA_A_ or glycine receptor activation (Figure 1). This, in turn, leads to membrane hyperpolarization in the form of inhibitory postsynaptic synaptic potentials. The upregulation of KCC2 during development is therefore necessary for the so-called “GABA switch”: namely, the conversion of the GABA_A_ receptor-mediated synaptic potential response from depolarizing to hyperpolarizing during early postnatal brain development [4,5]. Indeed, overexpression of KCC2 in immature neurons is sufficient to render GABA as an inhibitory neurotransmitter [6].

Prior to the developmental upregulation of KCC2, GABA_A_ receptor-mediated depolarization is essential for circuit assembly [7–9]. The GABA switch is then likely critical for cementing circuit formation, possibly by directing the formation of inhibitory synapses [10] and thereby establishing a proper excitatory/inhibitory balance in the brain. There are, however, regional differences in GABA inhibitory maturation during development. In general, evolutionarily older structures (i.e. the spinal cord and brainstem) show earlier increases in KCC2 mRNA expression as compared to “newer” structures (i.e. the cerebral cortex), paralleling the sequential maturation of neurons in these regions [11]. It must be noted, however, that there are potential mismatches between mRNA and protein expression, such that early increases in KCC2 mRNA may not necessarily translate into a functional excitatory to inhibitory switch [11]. Interestingly, KCC2, via its interaction with the cytoskeleton and independent of its ion transporting activity, also plays a critical role in the formation, maintenance, and regulation of excitatory glutamatergic synapses [12–14].

At this point, the reader must also be alerted to a controversy that arose over a decade ago regarding the possibility that the excitatory actions of GABA in immature neurons might have been the result of an experimental artifact. Indeed, Zilberter and colleagues suggested that the preferred energy sources utilized by immature neurons *in vivo* (i.e. lactate, pyruvate, and beta-hydroxybutyric acid), when used *in vitro* with glucose, do not lead to GABA-mediated excitation [15,16]. These findings were refuted by Ben-Ari and colleagues [17]. Nevertheless, definitive follow-up studies addressing these points are lacking [15].

Consistent with its critical role in synapse activity, the function of KCC2 is modulated via a number of regulatory signaling pathways, most prominently through phosphorylation [18]. For instance, it has been suggested that phosphorylation of key regulatory residues within the KCC2 molecule (i.e. T906 and T1007) by chloride-sensitive kinases, such as the WNK1-SPAK kinase complex, can reduce KCC2 function, which consequently regulates the inhibitory actions of GABA as a form of synaptic plasticity [19,20]. KCC2 activity via cell surface expression is also modulated by the activity of the metabotropic zinc-sensing receptor mZnR/GPR39 [21–23], which is activated by the release of zinc into the synapse from excitatory glutamatergic terminals [24]. This process is likely responsible for maintaining homeostatic plasticity during periods of increased excitatory drive, since impaired zinc-dependent regulation can lead to aberrant excitation and seizures [24]. In addition to epilepsy, KCC2 dysfunction has been implicated in schizophrenia, neuropathic pain, and autism, among other neurological disorders [25,26]. As such, KCC2 has been proposed as a viable therapeutic target in disorders associated with an imbalance in excitatory/inhibitory signals, which leads to circuit hyperexcitability [27,28].

**Figure 2.**
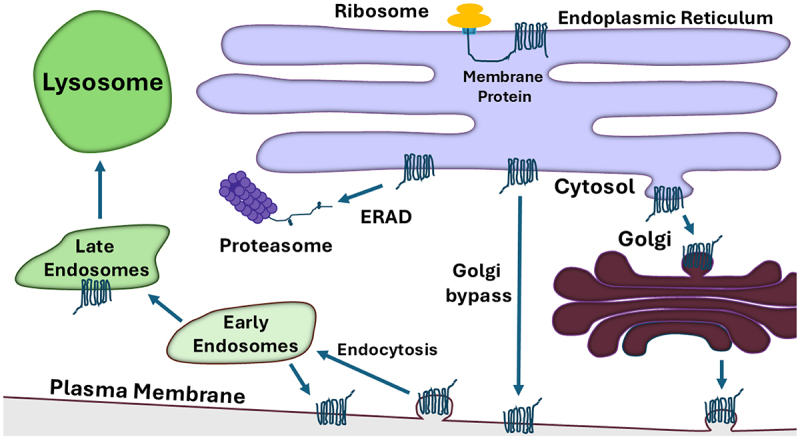
Membrane proteins, such as KCC2, are first synthesized by ribosomes on the endoplasmic reticulum (ER) surface and then translocate into the ER. Protein folding is initiated co-translationally but is completed post-translationally. If the native state and the proper post-translational modifications are appended, the proteins exits the ER for further modification in the Golgi or in some cases can proceed directly to the plasma membrane (via Golgi bypass). Misfolded proteins may instead be targeted for endoplasmic reticulum associated degradation (ERAD). Once at the plasma membrane, membrane proteins are internalized and can be routed for lysosome-dependent degradation or are recycled back to the membrane from early endosomes.

Also consistent with its critical role in synapse activity, the biogenesis of KCC2 is carefully monitored by cellular quality control pathways, which ensure that only properly folded and mature forms of the protein are expressed in neurons (Figure 2). The most common and earliest acting quality control step monitors the synthesis, folding, and post-translational modification of KCC2 in the endoplasmic reticulum (ER) [29,30]. During these early events, protein maturation is supervised by a series of molecular chaperones and chaperone-like lectins. Should KCC2 fail to attain its native structure and acquire critical disulfide bonds and N-linked glycans, these non-native KCC2 species can be retained in the ER, thus preventing their transport to the cell surface where a misfolded protein might otherwise have pathophysiological consequences [31,32]. In some cases, non-natively folded ion transporters – including misfolded forms of a KCC2 family member, the sodium-chloride co-transporter NCC – can be targeted to the ubiquitin-proteasome pathway, which destroys and thus prevents proteotoxic stress in the ER [33,34]. This degradative process is known as ER associated degradation (ERAD), and a vast collection of mutated forms of ion channels and transporters have been identified that are folding compromised and subjected to the ERAD pathway [35–37]. Nevertheless, a second line of protein quality control also exists if non-native proteins escape the ER: aberrant proteins that access later compartments of the secretory pathway (e.g. the Golgi apparatus, endosomes, or the plasma membrane) can also be selected and ubiquitinated. In these cases, the modified protein substrates are instead directed for lysosomal degradation [38,39].

In this article, we discuss in greater detail how KCC2 is synthesized, matures, and is transported to the cell surface. We also explore the consequences when *SLC12A5* harbors disease-causing mutations that result in folding defects, and we summarize a new strategy to predict the fate of folding compromised KCC2 mutants. In addition, we outline how natively folded and active forms of KCC2 are regulated by the ubiquitin-proteasome pathway, thereby establishing this pathway in both protein quality control as well as protein quantity control.

## KCC2 biogenesis and transport through the secretory pathway

KCC2 biogenesis in the secretory pathway begins when ribosomes synthesize a nascent polypeptide on the cytosolic face of the ER membrane (Figure 2). Like other members of the cation chloride co-transporter (CCC) family, the structure of KCC2 is elaborate, with 12 transmembrane domains and a large extracellular loop that is initially deposited into the ER lumen [40–42]. This loop contains multiple residues that facilitate protein folding and promote KCC2 maturation from the ER to the Golgi and on to the cell surface. For example, KCC2 has six sites for N-linked glycosylation (N283, N291, N310, N328, N338 and N339) that acquire core glycans (Glc_3_Man_9_GlcNAc_2_) in the ER [3,43]. As seen with other glycoproteins [44], the trimming of the terminal glucose residues on each core glycan results in the recruitment of protein folding chaperones and enzymes to KCC2. Upon removal of the final glucose and liberation from the folding machinery, natively folded KCC2 becomes encapsulated into COPII vesicles in the ER, which is required for transport to the Golgi, at which point complex glycans are added [31,45]. In contrast, if folding is delayed in the ER, the mannose core on the glycan is trimmed by ER chaperone-like mannosidases, which target non-native proteins for ERAD (see next section).

The stepwise acquisition of glycans from their core (i.e. ER-modified) to their complex (i.e. Golgi-modified) states allows one to monitor KCC2 maturation over time using western blot analysis [46]. These experiments were conducted in HEK293 cells and show that almost all core glycosylated KCC2 matures into its complex glycosylated state after eight hours. However, cell surface biotinylation experiments in HEK293 cells detected both core and complex glycosylated KCC2 at the cell surface. This result suggests the existence of a Golgi bypass pathway in which core glycosylated KCC2 is transported directly from the ER to the plasma membrane (Figure 2). While the functional significance of this pathway has yet to be determined, it is important to note that other transporters within the CCC family have also been reported to utilize the Golgi bypass pathway [47,48] as well as a voltage-gated potassium channel [15]. Furthermore, affinity purification experiments identified KCC2 in its core glycosylated state on neuronal cell membranes, suggesting that evidence for this pathway is not an artifact arising from the use of HEK293 cells [49]. Because KCC2 expression and function is developmentally regulated (see above), it is intriguing that members of the Golgi reassembly-stacking protein (GRASP) family, which have been implicated in Golgi bypass of membrane proteins [50,51], are upregulated and then re-localize to the plasma membrane during development [52]. Other data indicate that the oligomeric state of GRASP is regulated by phosphorylation, and re-localization to the ER is instead required for unconventional protein secretion [53]. In this case, however, the switch to GRASP-dependent Golgi bypass is ER stress-induced. In the future, it will be important to examine whether GRASP, or other factors linked to unconventional protein secretion (e.g. components of the autophagy machinery) [50,51], associate with or regulate KCC2 secretion.

It is also important to note that the Golgi bypass pathway can be induced by ER stress [50,51]. For example, both wild-type and a disease-associated mutated form of the cystic fibrosis transmembrane conductance regulator arrive at the cell surface in a Golgi-independent manner when host cells undergo ER stress [54]. Because various neuronal pathologies are associated with ER stress [55], it will also be important to examine if these conditions (e.g. stroke [56]) alter the trafficking itinerary of KCC2, which could readily be accomplished by measuring surface levels of EndoH-sensitive versus resistant forms of KCC2 in established models of stroke.

In addition to the six sites for N-linked glycosylation, KCC2 contains four cysteine residues (C287, C302, C322, and C331) that form disulfide bonds, which ultimately reside in the large extracellular loop. While mutating a single cysteine has no effect on KCC2 function, there was a > 75% reduction in KCC2 activity when triple and quadruple mutants were examined in HEK293 cells [57]. Interestingly, KCC2 cell surface expression was unaffected, indicating that disulfide bond formation, although critical for protein function, has no impact on intracellular transport to the cell surface.

**Figure 3.**
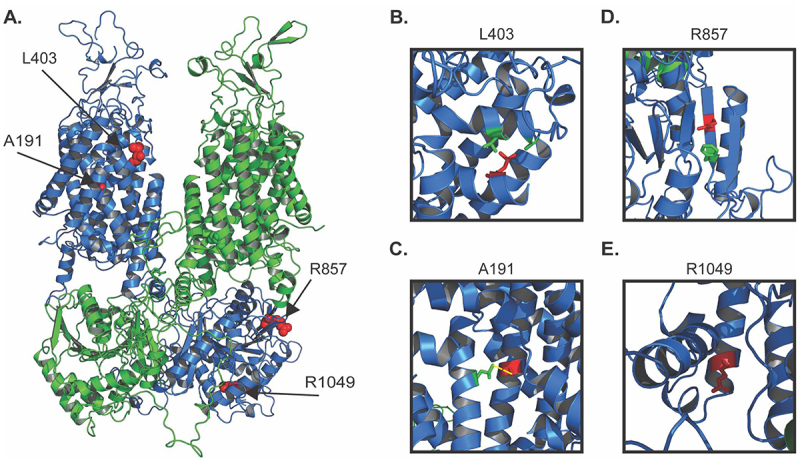
Structural view of natively folded and select folding-disrupting KCC2 variants. **A**. 3D homology model of dimeric KCC2 with chain A (green) and chain B (blue). KCC2 homology model was built from the KCC3 cryo-EM structure (PDB: 6Y5R) and obtained from SWISS-MODEL [41]. Four disease-linked mutations discussed in the text and Table I are depicted in surface representation and colored red. **B**. Residue L403 is depicted with its side chain shaded red and together with V123 and A127 in green. **C**. A191 is depicted in red and its measured proximity to L561(green) is shown as yellow-dashed line which denotes ~3.9 angstroms. **D**. R857 is depicted in red and F859 is in green. **E**. R1049 is depicted in red and is located at the end of an alpha helix near the C-terminal end of KCC2.

Another elaborate aspect of the KCC2 structure is the formation of a stable dimer in which the intracellular domains cross over one another to form a scissor conformation (Figure 3(A)) [40–42]. In this structure, the C-terminal domain (CTD) of one monomer interacts with the transmembrane domains (TMDs) of the neighboring monomer, and hydrophobic interactions between TMDs 11 and 12 further stabilize the protein in the dimeric state. Western blot analyses with neuronal lysates reported a correlation between the monomer:dimer ratio and brain development in mice, with an increase in dimer formation as development proceeds [58]. However, it is important to note that the extent to which the monomer:dimer ratio changes depends upon the region of the brain in which KCC2 was analyzed: the cortex experienced the most dramatic increase in dimer abundance, whereas the ratio was unchanged in the olfactory bulb. These data imply that while KCC2 dimerization may play a critical role in brain development, the mechanism of dimer assembly and the specific functional implications of these effects are unclear.

## KCC2 regulation and quality control

Like most proteins that are developmentally and/or functionally regulated, KCC2 acquires myriad post-translational modifications that control protein localization, stability, association with protein partners, and/or activity. For example, the exposure of mannose residues in KCC2 core glycans in the ER (also see previous section) may be sufficient to target the transporter for degradation via the ERAD pathway (Figure 2) [31]. To begin to explore whether non-native KCC2 species are selected for ERAD, we examined KCC2 biogenesis in the presence or absence of proteasome inhibitors in three models, including a heterologous KCC2 expression system in yeast and HEK293 cells along with primary rat neurons that natively express KCC2 [46]. Although overexpressed KCC2 was robustly degraded by ERAD in yeast, only a minor portion of KCC2 was targeted for ERAD in HEK293 cells. In contrast, endogenous KCC2 in primary rat neurons was completely stable, even after eight hours. These results indicate that KCC2 folds proficiently when expressed at native levels and in a native environment, but a variable fraction of the protein misfolds when overexpressed, especially in non-native cells.

Another hypothesis to explain these results is that yeast, HEK293 cells, and the brain express different levels of specific chaperones which are required for ER quality control/ERAD. In fact, prior work has noted that the ERAD pathway is hyperactivated in yeast compared to HEK293 cells [59]. In addition, while chaperone levels are generally unchanged across different human tissues, there is a notable decrease in the levels of HSPA1A (which encodes the major inducible Hsp70) in most brain regions in relation to most other tissues [60]. Moreover, HEK293 cells express higher levels of this Hsp70 isoform compared to other cell lines [61]. Based on the structure of KCC2 and the known function of HSPA1A, it is likely this chaperone interacts with the large, cytoplasmic region of KCC2 which forms the channel’s interwoven dimer [62]. In the future, a careful analysis of how altered levels of HSPA1A, as well as other chaperones, engage and regulate KCC2 stability is imperative.

## KCC2 regulation and quantity control

Another step at which KCC2 turnover occurs in the secretory pathway is at the plasma membrane, the location at which the protein may be subjected to endocytosis and, potentially, lysosome-mediated degradation (Figure 2). Indeed, KCC2 contains a di-leucine endocytic motif (L657 and L658) situated within the intracellular CTD that interacts with the AP2 receptor to trigger clathrin-mediated endocytosis [63]. Mutation of these residues to alanine results in the retention of KCC2 at the cell surface. In addition to this motif, the CTD contains multiple phosphorylation sites that also regulate KCC2 stability on the plasma membrane. For example, phosphorylation of S940 by protein kinase C (PKC) stabilizes KCC2 at the cell surface [64], while phosphorylation of Y1087 and Y903 by Src-family tyrosine kinases reduces plasma membrane stability [65]. As mentioned in the preceding section, T906 and T1007 are phosphorylated by the WNK1-SPAK kinase complex, which decreases KCC2 surface expression and thus mitigates its function [66]. This, in turn, reduces the ability of the cell to extrude chloride and renders GABA signaling excitatory. In fact, increased phosphorylation of T906 and T1007 in KCC2 is coincident with seizures when inhibitory neurotransmission is impaired [19,67]. Although other phosphorylation sites have been identified in KCC2, the impact of the majority of these on KCC2 trafficking, stability, and activity are uncharacterized [68,69].

Following endocytosis at the cell surface, membrane proteins may be routed from early recycling endosomes and then on to late endosomes, at which point they can then be subject to lysosome-dependent degradation [38] (Figure 2). Perhaps not surprisingly, several examples of KCC2 modulation via this pathway have been described. For example, the Cbl family of E3 ubiquitin ligases has been shown to trigger the modification and endocytosis of tyrosine kinases [70]. However, one member of this family, Cbl-b, supports KCC2 endocytosis by facilitating lysine-48-linked ubiquitination when this process was examined in a mouse spinal injury model [71]. Following injury, KCC2 ubiquitination by Cbl-b leads to a reduction in both KCC2 cell surface levels and overall expression, consistent with its internalization and degradation in the lysosome. A role for tyrosine kinase-dependent regulation was also seen following the induction of seizures in adult mice via the application of the muscarinic acetylcholine receptor agonist, pilocarpine [65]. This drug prompts phosphorylation of the aforementioned tyrosine residues (Y903 and Y1087) and triggers a swift lysosome-dependent decline in KCC2 levels. Similar observations were obtained under conditions of increased glutamatergic signaling by NMDA in rat hippocampal slices [72,73].

A lysosome- and proteasome-independent degradation pathway that down-regulates KCC2 levels has also been reported. In brief, a large section of the KCC2 C-terminus is cleaved by a calcium-activated protease, m-calpain [72,74]. This is the result of MAP kinase pathway activation and subsequent phosphorylation of the protease to initiate KCC2 destruction. Experiments in a rat model for epilepsy indicate that calpain-mediated cleavage reduces total KCC2 as well as cell surface KCC2 [74,75]. Moreover, administration of a pan-calpain inhibitor reduces the severity and incidence of seizures [76]. Similar findings were evident in a rat model for neuropathic pain [77]. Consequently, calpain-dependent cleavage of KCC2 contributes to neurological disease and could represent a promising target for therapeutic treatment. Nevertheless, experimental data are lacking to link calpain-mediated cleavage of KCC2 and subsequent ubiquitination and lysosomal degradation. As such, it appears, at least thus far, that both forms of regulation operate independently. For example, NMDA activation leads to the dephosphorylation of S940, causing KCC2 to be internalized from the cell surface [73]. Considering that calpain cleavage of KCC2 is also triggered in response to NMDA activation [78], there might be an overlap between the two regulatory mechanisms. Future experiments that explore this relationship, such as co-immunoprecipitation assays to assess the interaction between phosphorylated KCC2 and calpain after NMDA is activated, would be valuable.

An intriguing alternate form of KCC2 down-regulation stems from studies undertaken to explore the effects of anesthesia. Although time-dependent recovery from anesthesia was generally considered to arise naturally, it now appears that recovery can come about via an active process. Notably, recent work implicates ubiquitin-dependent degradation of KCC2 in the thalamus as a trigger for recovery from anesthesia [79]. More specifically, using both animal and *in vitro* tools, KCC2 phosphorylation at T1007 was found to favor the interaction between KCC2 and a component in an SCF-type E3 ubiquitin ligase, Fbxl4. This event takes place in the ventral posteromedial nucleus of the thalamus, which is one of two areas comprising the sensory thalamus. In a follow-up paper, the same team showed that both the AAA-ATPase, known as VCP/p97, which extracts ERAD substrates from the ER membrane for delivery to the ubiquitin-proteasome system, as well as FAF1, which is a ubiquitin-binding VCP co-factor, are required for KCC2 proteolysis. This phenomenon was demonstrated using both genetic and pharmacological methods [80].

Zinc is a neuromodulator that is co-released with glutamate from excitatory terminals in vast areas of the brain, most prominently in the hippocampus, cerebral cortex, amygdala, and auditory brain stem [24]. Synaptic zinc regulates the function of a large number of neurotransmitter receptors, most notably the glutamatergic NMDA receptor [81]. In addition, zinc directly and specifically activates a metabotropic, G-protein linked receptor: mZnR/GPR39 [82]. The activation of mZnR/GPR39 by synaptic zinc leads to SNARE-dependent upregulation of KCC2 at the postsynaptic membrane, which results in increased chloride transport and enhanced inhibitory tone [21–23]. This form of homeostatic plasticity is important during periods of increased excitatory activity, as the absence of mZnR/GPR39 renders animals highly susceptible to fulminant seizure activity [83]. Of interest, intracellular, rather than extracellular zinc, can block KCC2 function [84].

Finally, it is noteworthy to mention the existence of KCC2 pharmacological stabilizers, including putative KCC2 enhancers/activators, such as CLP257 and its brain penetrant precursor CLP290, which act by increasing surface expression of the transporter [85]. However, the specificity of CLP257 action has been questioned [86], although these contradictory findings have also been refuted [87].

## The fates of disease-causing KCC2 mutants

In principle, folding-compromised plasma membrane-targeted proteins in the secretory pathway can be: (i) stable but trapped in the ER, (ii) destroyed by the ERAD pathway, (iii) retained in the Golgi or in plasma membrane-directed vesicles, (iv) exhibit rapid retrieval from the cell surface for lysosome-dependent degradation, or (v) simply lack activity at the plasma membrane (Figure 2). Perhaps, the best example in which each of these fates has been shown for diverse disease-associated mutants is the Cystic Fibrosis Transmembrane conductance Regulator (CFTR). In many cases, a single mutation may result in more than one of these fates, e.g. CFTR alleles exist that are both targeted for ERAD and – if the folding of the mutant is corrected with pharmacological chaperones – exhibit a lower open probability (Po) for chloride transport [88]. The importance of binning disease-associated mutants into these classes is underscored by the generation of drugs developed to “correct” protein folding or to increase Po [89]. Applying these specific drugs to patients requires preexisting knowledge of the defects associated with an individual’s mutant allele.

As outlined in the Introduction, mutations in KCC2 have been identified in multiple neurodevelopmental disorders, including schizophrenia, autism spectrum disorders, and epilepsy [37,43]. However, until recently, a relatively small number of KCC2 mutant alleles had been characterized (Table 1). Prior work established that six disease-causing mutants exhibited lower activity (L288H, L403P, M415V, G528D, R952H, and R1049C), and amongst five of these which were examined, three were depleted at the cell surface (L288H, L403P, and R952H) [90–92]. The molecular basis of this phenomenon was not examined. In turn, two of the disease-associated mutants (R952H and R1049C) alter phosphorylation of the cell surface-stabilizing serine at position 940 [95]. Quite recently, yet another mutant was identified (R231H), which results in reduced residence at the cell surface and thus lower activity, and is linked to migrating focal seizures in infancy [96].

**Table 1.** Disease-associated KCC2 mutants, pathogenicity predictions, and a summary of experimental findings. The indicated mutants are ranked from highest Rhapsody score (top) to lowest Rhapsody score (bottom). The higher the Rhapsody score, the more likely the indicated mutation results in catastrophic effects on protein maturation. AlphaMissense scores were also determined and rank the mutations from most deleterious (highest score) to least deleterious predictions (lowest score). In contrast, ESM1b ranks the mutants such that the lowest scores indicate the mutants that are predicted to be the most pathogenic.

Mutation	Rhapsody Score	Alpha-Missense Score	ESM1b Score	Experimental Findings	Citations
L403P	0.95	0.9964	−12.855	Decreased activity Decreased cell surface expression Defective maturation	[62,90]
R857L	0.91	0.9832	−12.462	Defective maturation Decreased cell surface expression	[62]
A191V	0.88	0.9966	−14.452	Unaffected activity Unaffected protein expression and cell surface expression	[62,91]
M415V	0.83	0.9893	−11.687	Decreased activity Unaffected protein expression and cell surface expression	[62,91]
R1049C	0.78	0.8946	−11.731	Decreased activity Unaffected cell surface expression and overall protein expression Decreased S940 phosphorylation	[62,92,93]
R1048W	0.68	0.8703	−11.019	Uncharacterized	[93]
L288H	0.61	0.7644	−13.608	Decreased activity Decreased protein expression and cell surface expression	[90]
G528D	0.53	0.9736	−15.032	Decreased activity Decreased protein expression and cell surface expression	[90]
S376L	0.53	0.2231	−4.791	Uncharacterized	[94]
W318S	0.51	0.7168	−11.257	Uncharacterized	[91]
S323P	0.36	0.1533	−7.552	Unaffected activity Unaffected protein expression and cell surface expression	[91]
R952H	0.31	0.0675	−6.487	Decreased activity Decreased cell surface expression Decreased S940 phosphorylation	[62,92,93,95]

To expand our knowledge of the defects associated with disease-associated KCC2 alleles, we recently examined the biogenesis of six KCC2 variants in a HEK293 cell model [62], as discussed earlier. The advantage of this model over others is that the maturation of KCC2 from the ER to the Golgi apparatus can be measured by visualizing the acquisition of Golgi-dependent outer-chain glycans [46]. Surprisingly, none of the KCC2 variants were subject to ERAD, yet the most folding compromised mutant (see below) was simply held in the ER and failed to traffic beyond this compartment (L403P) [62]. Further analyses in HEK293 cells found that two of the KCC2 mutants were subject to lysosome-mediated degradation (L403P and R857L). Several of the variants also exhibited decreased residence on the plasma membrane in cell surface biotinylation assays (R952H, L403P, and R857L). Interestingly, the species at the plasma membrane had both mature/complex glycans, which had been appended in the Golgi, as well as immature/ER appended glycans. The latter species is likely due to utilization of the Golgi bypass pathway.

## Can the fate of disease-causing KCC2 mutants be predicted?

To date, it is impossible to predict the severity of KCC2 mutant alleles. This hinders the ability of clinicians and genetic counselors to anticipate future treatments and disease outcomes. Therefore, we were curious whether it was possible to predict the relative inability of disease-associated KCC2 mutants to exit from the ER, access Golgi-resident glycosyltransferases, and traffic to and function at the cell surface [62]. To this end, pathogenicity scores for 12 disease-associated KCC2 mutants were determined using three established computational methods: Rhapsody, AlphaMissense, and ESM1b (Table 1) [97–100]. Rhapsody is a machine learning program that utilizes data on sequence coevolution along with structure- and dynamics-based features [97,101], whereas AlphaMissense capitalizes on structural information from AlphaFold and evolutionary conservation to predict mutation-specific pathogenicity [102]. ESM1b is a protein language model that “learns” how an amino acid sequence establishes secondary protein structures, long-distance interactions between sequences, post-translational modifications, and putative protein–protein binding sites [100]. Although ESM1b and AlphaMissense can provide a greater breadth of predictions, this comes with the potential of lost accuracy. For example, even though AlphaMissense does include structural information, it cannot account for proteins that assemble into multimers, such as the interwoven dimer of KCC2 [103]. By contrast, Rhapsody can account for multiple chains by using an anisotropic network model [98]. Therefore, we reasoned that Rhapsody provides the most evidence-based predictive scores for KCC2 relative to the other platforms. Indeed, a clear correlation between the Rhapsody pathogenicity score and the relative magnitude of a maturation defect for a KCC2 mutant was uncovered [62]. Simply put, the higher the Rhapsody score, the more likely the mutant protein exhibits a maturation defect and lacks Golgi-modified glycans (Table 1). The correlation was particularly clear for Rhapsody scores > 0.9. Since these values are considered “highly deleterious,” and because the maturation of mutant KCC2 species was unaffected if they had lower (i.e. less pathogenic) scores, we propose that the ER quality control machinery retains only the most severely folding-compromised variants.

When mapping a subset of the variants onto a homology model of KCC2, it’s clear the mutations with Rhapsody scores >0.9 are in structurally critical locations (Figure 3(A-E)). For instance, L403P (Rhapsody score = 0.95) is located within a region of closely packed α-helices (Figure 3(B)), where mutation to a chain-breaking proline will have a substantial impact on the local folding environment. L403P is therefore likely to disrupt stabilizing hydrophobic interactions between the leucine with both V123 and A127 (green side chains, Figure 3(B)). By contrast, A191V (Rhapsody score = 0.88, which is somewhat lower) occurs within transmembrane helix 3 and is in proximity to leucine 561 in a neighboring helix. Substitution to valine, in the disease-linked allele A191V, would likely maintain proper hydrophobic interactions due to valine’s nonpolar side chain. Although the valine side chain occupies greater volume, this should not result in a steric disruption of the α-helix packing and should only reduce the interaction with L561 (green side chain) from ~3.9 to 2.7 angstroms (Figure 3(C), yellow dashed line). Indeed, to date, no defects associated with this allele have been reported (Table I). In contrast, the R857L mutation (Rhapsody score = 0.91) is within a parallel β-sheet on the cytosolic face of KCC2 (Figure 3 3(D)). The exchange of the polar side chain of arginine for a hydrophobic side chain likely disrupts the β-sheet by weakening side-chain hydrogen bonds, salt bridges, and/or a cation-π interaction with F859 (Figure 3 3(D), green side chain). It is perhaps no surprise that this allele is maturation-defective. However, R1049C (Rhapsody score = 0.78, which is much lower), is located at the end of an alpha helix near a turn into an unresolved/unstructured loop (Figure 3(E)). Even though mutation to cysteine removes a positive charge, the residue faces away from the central core of the helix and should be tolerated. These observations are in line with the mutation having only a modest impact on KCC2 maturation. Instead, this allele likely exerts its effect on KCC2 and neuronal biology through a different mechanism, such as altered ion transport activity, which has been shown experimentally (see Table I).

In contrast to the predictive power of Rhapsody to identify ER retained mutants, Rhapsody was instead able to predict the relative efficiency of ERAD targeting when mutations in a renal potassium channel, known as ROMK, were examined [104]. Therefore, Rhapsody predicts distinct fates for misfolded forms of two different proteins. Because the KCC2 mutants were largely ERAD-resistant, at least in HEK293 cells (see above), either non-native disease-associated forms of KCC2 lack a structural defect required for ERAD targeting, or HEK293 cells lack a critical quality control factor that selects structurally compromised KCC2 variants for delivery to the ERAD machinery. Future work will investigate these hypotheses.

## Conclusions

In this article, we have outlined the pathways controlling the synthesis, maturation, and trafficking of KCC2 through the secretory pathway along with some of the major regulatory checkpoints the transporter encounters along the way. We next described the effects on KCC2 biogenesis when disease-associated point mutations are present. While the mutant proteins are relatively stable in mammalian cells and have escaped the ERAD pathway, we note that their acquisition of complex glycans in the Golgi – which serves a readout for cell surface localization – is not only differentially affected, but this outcome can be predicted by a machine learning tool.

With the ever-growing list of mutations identified in disease-linked proteins, such as KCC2, along with improved screening and diagnostic methods, pathogenicity predictors, such as Rhapsody, will become an increasingly valuable resource. Therefore, future efforts will be required to expand the number of characterized KCC2 variants, as this will improve the power of computational predictions of KCC2 maturation. Subsequent work may alternatively reveal that some KCC2 mutants are targeted for premature degradation in the ER via ERAD or in the lysosome. In parallel, new and improved pathogenicity programs will undoubtedly be developed in the coming years. It will prove especially instructive if a platform is able to distinguish between those mutants that are unable to transit to and mature in the Golgi, those which are targeted for ERAD, those which are improperly regulated, and those which traffic through the secretory pathway but might lack ion transporting activity at the cell surface. The development of this program would greatly facilitate the discovery of drugs that rectify specific phenotypic outcomes, and – once patient registries are developed to better report on disease severity – lead to personalized treatments for individuals afflicted with KCC2-associated diseases.

## Data Availability

Data sharing is not applicable to this article as no data were created or analyzed in this study. The authors confirm that the data supporting the findings of this study are available within the article.

## CRediT

MK: Conceptualization; Visualization; Writing-Original Draft; Writing-Review and Editing

EA: Conceptualization; Funding Acquisition; Writing-Original Draft; Writing-Review and Editing

JLB: Conceptualization; Funding Acquisition; Project Administration; Supervision; Writing-OriginalDraft; Writing-Review and Editing
